# Interleukin-1β Downregulates RBP4 Secretion in Human Adipocytes

**DOI:** 10.1371/journal.pone.0057796

**Published:** 2013-02-27

**Authors:** Primoz Kotnik, Michaela Keuper, Martin Wabitsch, Pamela Fischer-Posovszky

**Affiliations:** 1 Division of Pediatric Endocrinology and Diabetes, Department of Pediatrics and Adolescent Medicine, Ulm University, Ulm, Germany; 2 Department of Pediatric Endocrinology, Diabetes and Metabolism, University Children’s Hospital, UKC Ljubljana, Ljubljana, Slovenia; Facultad de Medicina, Universidad Autonoma Madrid, Spain

## Abstract

**Aims/hypothesis:**

The excessive accumulation of adipose tissue in the obese state is linked to an altered secretion profile of adipocytes, chronic low-grade inflammation and metabolic complications. RBP4 has been implicated in these alterations, especially insulin resistance. The aim of the present study was to determine if a local inflammatory micro-environment in adipose tissue regulates RBP4 expression and secretion.

**Methods:**

Human SGBS and primary adipocytes cultured with conditioned media from human THP-1 macrophages were used as an *in vitro* model for adipose inflammation. Adipocytes were exposed to recombinant TNF-α, IL-1β, IL-6 or IL-8. In addition, coexpression of IL-1β and RBP4 was measured in adipose tissue samples from 18 healthy females. RBP4 expression was studied by quantitative PCR and ELISA.

**Results:**

RBP4 mRNA expression and secretion was significantly reduced upon incubation with macrophage-conditioned media in SGBS adipocytes and human primary adipocytes. Out of several factors studied we identified IL-1β as a new factor regulating RBP4. IL-1β significantly downregulated RBP4 mRNA and secretion in a time- and dose-dependent manner. IL-1β mediated its inhibitory effects on RBP4 expression via IL-1 receptor and NF-κB, as incubation with the IL-1 receptor blocking antibody and the NF-κB inhibitors CAPE and SC-514 reversed its effect. Most interestingly, RBP4 mRNA was negatively correlated with IL-1β mRNA in subcutaneous adipose tissue.

**Conclusions:**

Adipose tissue inflammation as found in the obese state might lead to a downregulation in local RBP4 levels. IL-1β was identified as a major factor contributing to the decrease in RBP4. The increase in circulating RBP4 that often precedes the development of systemic insulin resistance is most likely unrelated to inflammatory processes in adipose tissue.

## Introduction

Adipose tissue has a major role in the regulation of systemic metabolism by secreting numerous factors, collectively called adipokines that act both locally and on distant tissues [1]. Excess accumulation of adipose tissue, especially visceral adipose tissue, is linked to an altered secretion pattern of adipocytes and to serious metabolic complications, of which systemic insulin resistance is of pivotal importance [2], [3]. Obesity has been linked to accumulation of macrophages in adipose tissue, pro-inflammatory activation of endothelial cells and consequently to a state of chronic low grade inflammation [4]. On the other hand, loss of adipose tissue is associated with a favorable adipokine profile, a decrease in adipose tissue inflammation, and an improved systemic insulin sensitivity [5]. Altogether, it is widely recognized that there is a causal link between dysfunction of adipocytes, chronic low-grade inflammation and metabolic consequences of obesity, especially insulin resistance [1].

RBP4 is a lypocalin protein transporting retinol in the circulation [6]. It is produced in the liver and also in mature lipid-laden adipocytes [7]. As an adipokine, it was first associated with the development of systemic insulin resistance in several mouse models [8]. In humans, adipose tissue and circulating RBP4 levels are associated with obesity, insulin resistance, type 2 diabetes and components of the metabolic syndrome [9], [10], [11], although it should be emphasized that these associations were not shown consistently [12]. Obesity-related adipose tissue inflammation was linked to increased adipose tissue RBP4 expression, which positively correlated with expression of inflammatory markers in human adipose tissue [13]. Circulating RBP4 levels, however, were not related to adipose tissue inflammation [13]. In contrast, the expression of RBP4 in human adipocytes was unexpectedly decreased by the inflammatory cytokine TNF-α [14].

The aim of the present study was to determine if an inflammatory micro-environment in adipose tissue might influence the production of RBP4 in human adipocytes. To this end, we used an *in vitro* model of inflamed adipose tissue, i.e. SGBS adipocytes cultured with conditioned media from human THP-1 macrophages. We furthermore aimed at identifying single factors regulating the expression of RBP4.

## Materials and Methods

### Human Subjects

Subcutaneous adipose tissue was collected from 18 healthy women (age 38.6±10.1 years; BMI 21–66 kg/m^2^) undergoing plastic surgery. Patients with malignancies were excluded from the study. Three adipose tissue samples from non-obese women (age 40.2±9.3 years; BMI 22–27 kg/m^2^) were used for the preparation of primary preadipocytes. All procedures in experimental subjects were performed in accordance with the Declaration of Helsinki guidelines and were approved by the ethics committee of the University of Ulm. Written informed consent was obtained from all subjects.

### Cell Culture

Human primary preadipocytes were prepared by collagenase digestion from subcutaneous adipose tissue of 3 healthy women using a previously described protocol [15]. SGBS preadipocytes were cultured as previously described [16]. Adipogenic differentiation of human primary and SGBS preadipocytes was induced in serum-free DMEM/F12 medium supplemented with 10 µg/ml iron-poor transferrin, 10 nM insulin, 200 pM thyroid hormone, 0.1 µM cortisol and for the first four days 2 µM rosiglitazone, 250 µM isobutylmethylxanthine and 25 nM dexamethasone.

THP-1 cells (ATCC, Wesel, Germany) were cultured in RPMI medium containing non-essential amino acids, L-glutamine, sodium pyruvate, antibiotics and 10% fetal bovine serum. Differentiation into macrophages was induced by 125 ng/ml phorbol myristate acetate for 48 hours. MacCM was collected after additional 48 hours of incubation in serum-free basal medium containing 0.5% BSA and cleared by centrifugation. MacCM were pooled from 5 independently performed experiments and then used for experiments. Cytokine concentrations in MacCM were determined by Bio-Plex system (Bio-Rad Laboratories, Hercules, CA, USA) and a commercially availably ELISA kit (Immunodiagnostik, Bensheim, Germany) and were as follows: TNF-α (39 ng/ml), IL-1β (390 pg/ml), IL-6 (2.1 ng/ml), IL-8 (55 ng/ml) and RBP4 (below detection limit).

### Treatments

Adipocytes were used for experiments on day 9 of adipogenic differentiation. For dose response experiments cells were washed in PBS and then treated in serum-free DMEM/F12 medium supplemented with 10 µg/ml iron-poor transferrin, 10 nM insulin, 200 pM thyroid hormone, 0.1 µM cortisol with in increasing doses of MacCM (5, 10, 20, 50%) or respective doses of vehicle (RPMI +0.5% BSA) for 48 hours. For kinetics, cells were treated for 6, 24, 48, and 72 hours with either 10% MacCM or 10% vehicle.

Cells were also treated with TNF-α (100 ng/ml), IL-1β (0.05, 0.5, 5, 50 ng/ml), IL-6 (50 ng/ml), and IL-8 (50 ng/ml) for 48 hours. To determine time-dependent effects SGBS adipocytes were treated with 5 ng/ml IL-1β for 6, 24, 48 and 72 hours.

To determine mechanisms of IL-1β action, adipocytes were treated with IL-1 receptor type 1 neutralizing antibody (IL-1R1 Ab) (100 µg/ml) or NF-κB inhibitors caffeic acid phenethyl ester (CAPE) (1 µg/ml) or SC-514 (100 µM) with or without IL-1β (0.5 ng/ml).

### Expression Analysis of mRNA by Quantitative Real Time PCR (qRT-PCR)

Total RNA was isolated from adipocytes or adipose tissue using RNeasy Lipid Tissue Kit (Qiagen, Hilden, Germany) following manufacturers instructions. cDNA was reversely transcribed using Superscript II reverse transcriptase (Invitrogen, Darmstadt, Germany). Gene expression was determined by qPCR (LightCycler, Roche, Mannheim, Germany). Intron-spanning primers were RBP4-fw: 5′-TAG CCT CCT TTC TCC AGA AAG GAA ATG ATG AC-3′, RBP4-rev: 5′-GGT TTC TTT CTG ATC TGC CAT CGC AGT AAC C-3′; succinate dehydrogenase (SDHA)-fw: 5′-CAT GCT GCC GTG TTC CGT GTG GG-3′; SDHA-rev: 5′-GGA CAG GGT GTG CTT CTT CCA GTG CTC C-3′; adiponectin-fw: 5′-GGC CGT GAT GGC AGA GAT-3′; adiponectin-rev: 5′-CCT TCA GCC CGG GTA CT-3′; peroxisome proliferator-activated receptor gamma (PPARγ)-fw: 5′-GAT CCA GTG GTT GCA GAT TAC AA-3′; PPARγ-rev: 5′-GAG GGA GTT GGA AGG CTC TTC-3′; CCAAT-enhancer-binding protein alpha (C/EBPα)-fw: 5′-GAC CCT CAG CCT TGT TTG TAC TGT ATG CC-3′; C/EBPα-rev: 5′-TTT GGA AAG CTT GTC ATA ACT CCG GTC CC-3′; CCAAT-enhancer-binding protein beta (C/EBPβ)-fw: 5′-CCG CCC GTG GTG TTA TTT AAA GAA GAA ACG TC-3′; C/EBPβ-rev: 5′-GCC CGT AGG AAC ATC TTT AAG CGA TTA CTC AG-3′. Data was analyzed by a comparative 2−^ΔΔCT^ method [17].

### RBP4 Enzyme-linked Immunosorbent Assay (ELISA)

Medium supernatants were collected and stored at −80°C until further analysis. RBP4 levels were determined by ELISA (Immunodiagnostik, Bensheim, Germany), according to the manufacturers instructions. Sensitivity of the test was 0.9 µg/l and inter- and intra-assay confidence values (CV) were 5 and 9.8% respectively.

### Determination of Adipogenic Differentiation Rates

The number of morphologically differentiated adipocytes was determined by direct microscopic counting using a net micrometer. Three independent experiments performed in triplicate were analyzed.

### Determination of Adipocyte Triglyceride Content

*In vitro* differentiated SGBS adipocytes were incubated in insulin-free medium supplemented with 10% MacCM for 48 h. Cellular triglyceride contents were measured using a commercially available triglyceride assay kit (Sigma-Aldrich, Munich, Germany).

### Lipolysis Assay

*In vitro* differentiated SGBS adipocytes were incubated in insulin-free medium supplemented with 10% MacCM for 48 h. Cell-free supernatants were harvested by centrifugation and glycerol content was determined using the Free Glycerol Assay Kit (Sigma-Aldrich, Munich, Germany) following the manufacturer’s protocol.

### Statistical Analysis

Data from 3 or more independent experiments were expressed as mean ± SEM. When applicable, Student’s *t*-test or ANOVA and post hoc Dunnetts test were used for comparison between groups. Statistical analysis was performed by GraphPad Prism version 5.0 (GraphPad, San Diego, USA). P-value <0.05 was considered statistically significant.

## Results

### Macrophage-secreted Factors Decrease RBP4 Expression in Human Adipocytes

**Figure 1:**
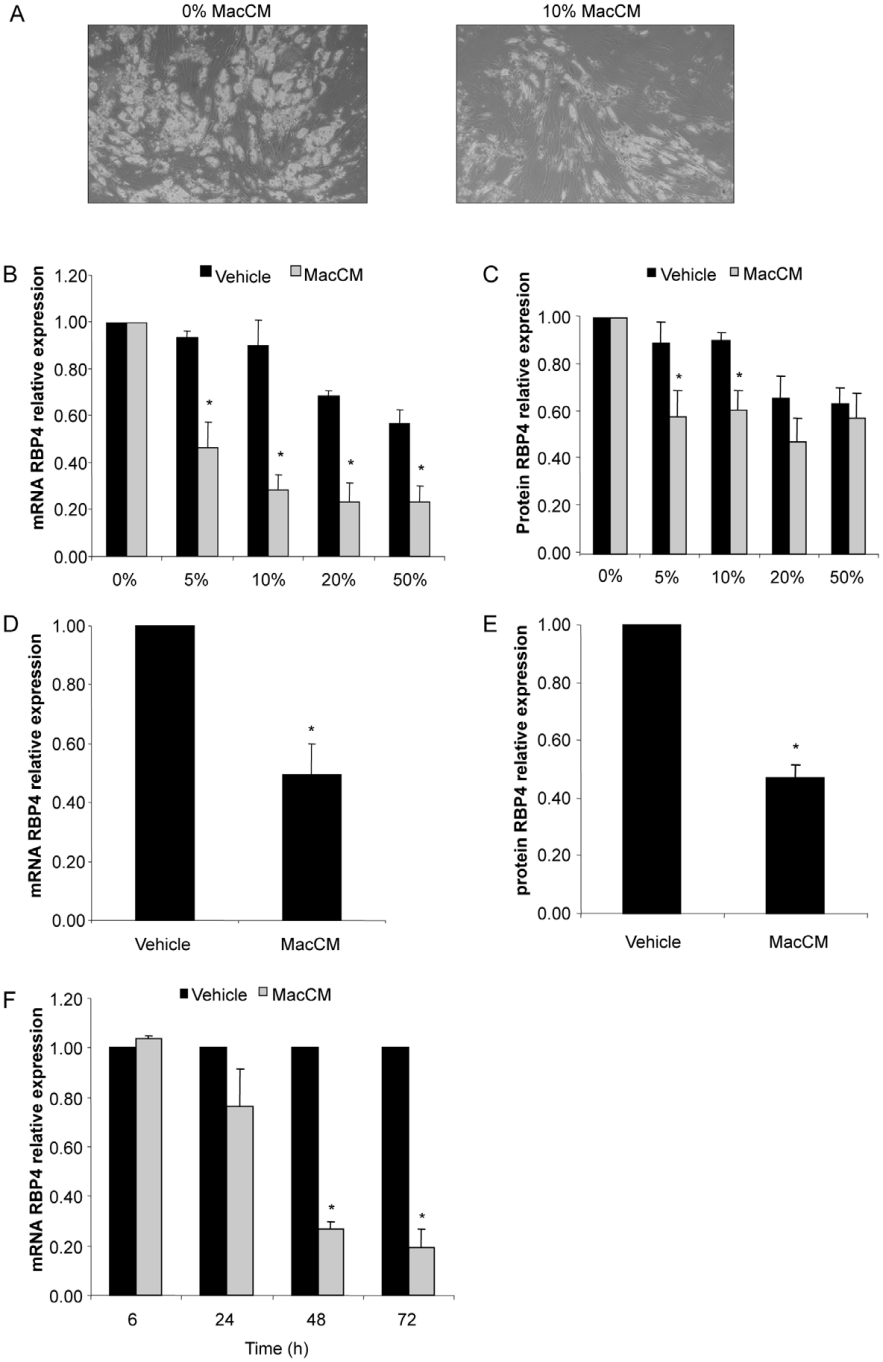
Macrophage-secreted factors decrease RBP4 production. SGBS adipocytes (A, B, C) were treated with increasing concentrations of MacCM (5, 10, 20, 50%) or the corresponding vehicle control. (A) Representative pictures of cells treated for 48 hours with 10% of MacCM. (B) RBP4 mRNA expression was measured by quantitative PCR. RBP4 expression was normalized to succinate dehydrogenase complex subunit A (SDHA) and related to medium control using 2^−ΔΔCT^ method. Data are presented as mean+SEM of three independent experiments. *p<0.05 (treatment vs. vehicle). (C) Accumulation of RBP4 in the medium supernatant was measured by ELISA. Measurements were related to untreated medium control. Data are presented as mean+SEM of three independent experiments. *p<0.05 (treatment vs. vehicle). (D, E) Human primary adipocytes obtained from three different donors were treated with 10% of MacCM or vehicle. (D) mRNA expression and (E) secretion of RBP4 was performed as described above. *p<0.05 (treatment vs. vehicle). (F) SGBS adipocytes were treated with 10% MacCM or vehicle for 24, 48, and 72 hours. mRNA expression of RBP4 was measured by quantitative PCR. RBP4 expression was normalized to SDHA and related to medium control at 0 hours using 2^−ΔΔCT^ method. *p<0.05 (treatment vs. vehicle).

To determine the effect of an inflammatory micro-environment on the expression of RBP4 in human fat cells, SGBS adipocytes were exposed to macrophage-conditioned media (MacCM) obtained from human THP-1 macrophages. Morphologically, we observed the previously described delipidation most likely caused by lipolysis [18], [19] (Figure 1A). Indeed adipocytes treated with 10% MacCM carried significantly less lipids compared to vehicle treated adipocytes (0.40±0.02 vs. 0.60±0.05 mg triglyceride/mg protein, respectively; p<0.05). In addition, there was accumulation of glycerol in media of MacCM-treated cells (32.7±8.7 vs. 23.3±6.9 µM; p<0.05), indicating increased lipolysis in MacCM adipocytes. The delipidation was accompanied by a decrease in C/EBPα and PPARγ expression (data not shown).

RBP4 mRNA expression was decreased by MacCM in a dose-dependant manner (Figure 1B). Already 5% of MacCM caused a downregulation of RBP4 mRNA to 46±11% of the vehicle. This effect was further increased by higher concentrations of MacCM to a maximum of 23±8% of the vehicle by medium with 20% MacCM. This finding was reflected on the protein level (Figure 1C), for example the accumulation of RBP4 in the medium supernatant was inhibited to 58±11% of the vehicle at 5% MacCM. The inhibition of RBP4 expression was time-dependent with a maximal effect at 72 hours with 10% MacCM (Figure 1F).

Starting at a concentration of 20%, the corresponding vehicle control also showed an inhibitory effect on RBP4 mRNA expression and secretion most likely due to dilution of the adipogenic basal medium and an increase in BSA concentration.

To demonstrate the physiological relevance of our findings we also tested the effect of MacCM on human primary *ex vivo* differentiated adipocytes obtained from three different donors. Comparable to SGBS adipocytes, we detected a downregulation of RBP4 mRNA expression (Figure 1D) and secretion (Figure 1E) to 49±9% of the vehicle by 10% MacCM.

This set of experiments clearly demonstrates that macrophage-secreted factors cause an inhibition of RBP4 production in human fat cells.

### IL-1β, but not IL-6 and IL-8 Inhibits RBP4 Expression in Human Adipocytes

**Figure 2:**
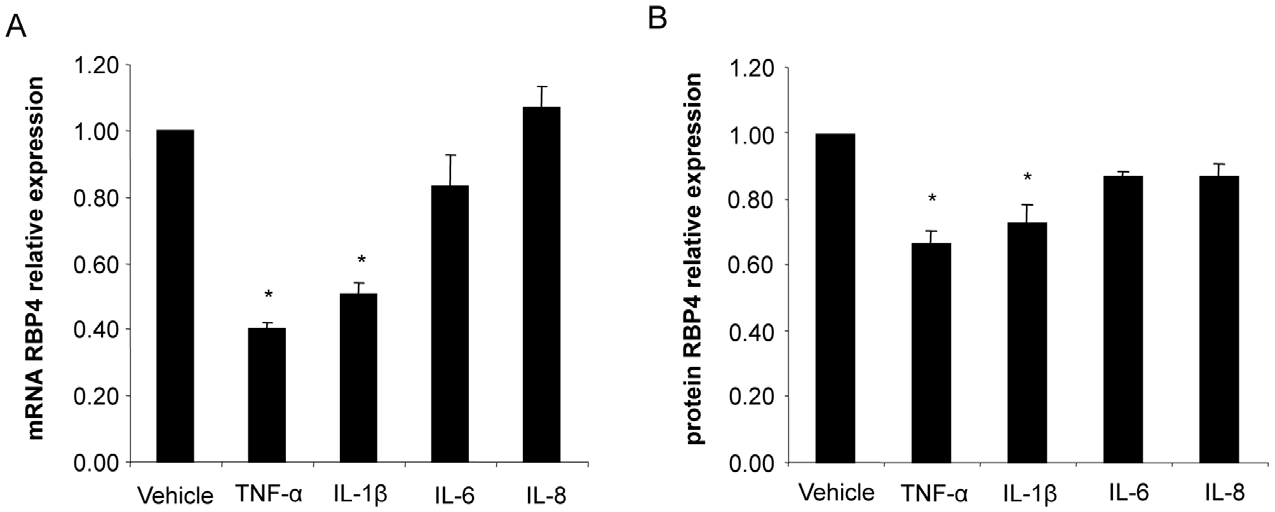
Regulation of RBP4 production by selected cytokines from MacCM. SGBS adipocytes were treated with TNF-α, IL-1β, IL-6 or IL-8 for 48 hours. (A) mRNA expression of RBP4 was measured by quantitative PCR. RBP4 mRNA expression was normalized to succinate dehydrogenase complex subunit A (SDHA) and related to medium control using 2^−ΔΔCT^ method. Data are presented as mean+SEM of three independent experiments. *p<0.05 (treatment vs. vehicle). (B) Accumulation of RBP4 in the medium supernatant was measured by ELISA. Measurements were related to untreated medium control. Data are presented as mean+SEM of three independent experiments. *p<0.05 (treatment vs. vehicle).

We next wanted to identify the factors responsible for the downregulation of RBP4. The typical macrophage-associated cytokines TNF-α, IL-1β, IL-6, and IL-8 were highly abundant in our conditioned media [19]. We therefore tested if these cytokines interfere with RBP4 production. As previously described by Sell et al. [14], TNF-α caused a robust downregulation of RBP4 production to 40±2% of the vehicle on the mRNA level (Figure 2A) and to 67±4% of the vehicle on the protein level (Figure 2B). Interestingly, we discovered that IL-1β was almost equally efficient in decreasing RBP4. 50 ng/ml IL-1β inhibited RBP4 mRNA to 51±3% of the vehicle and its secretion into the culture medium to 73±5% of the vehicle. Both IL-6 and IL-8 did not significantly alter RBP4 production.

### IL-1β Inhibits RBP4 Production in a Dose- and Time-dependent Manner

**Figure 3:**
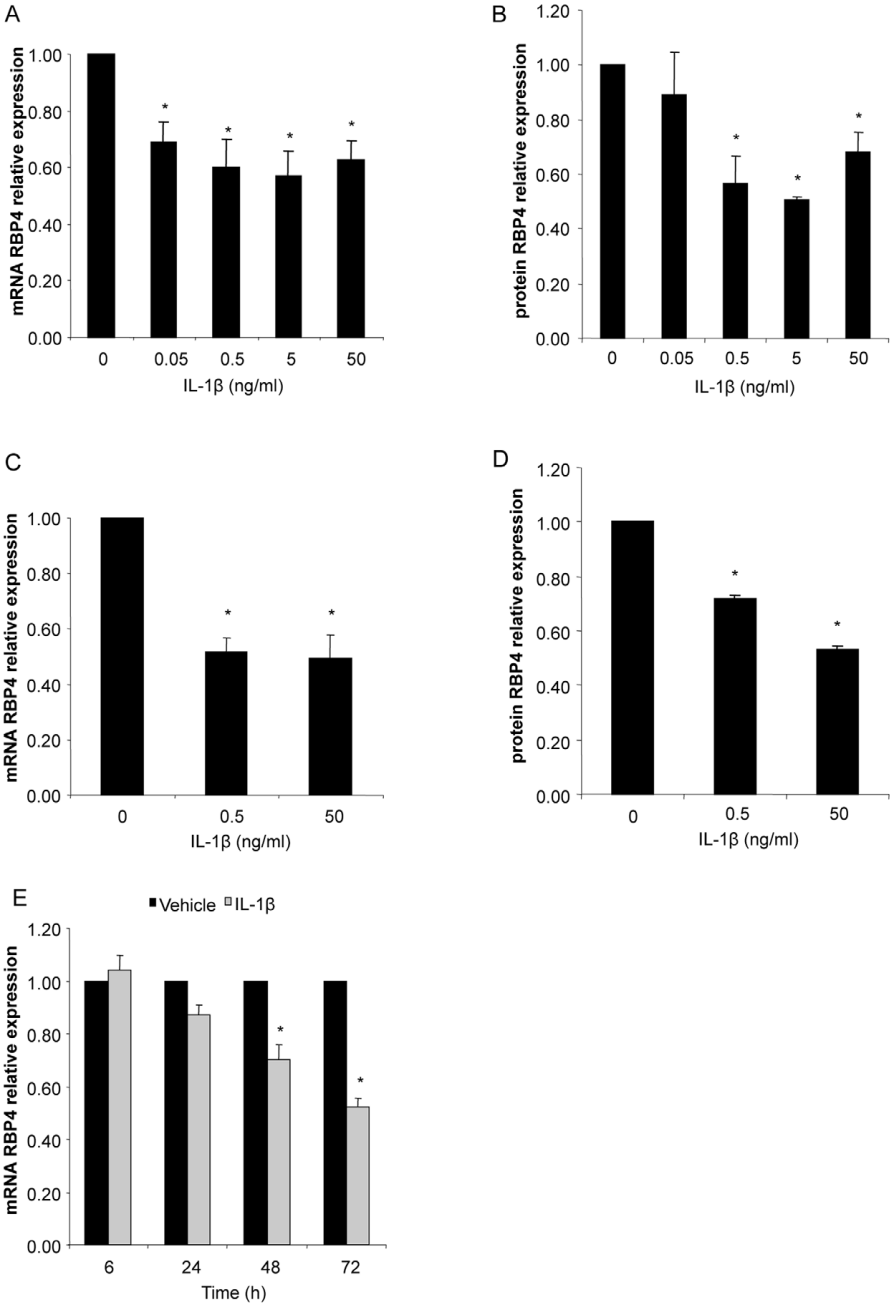
IL-1β decreases RBP4 production. SGBS adipocytes were treated with increasing doses of IL-1β (0.05, 0.5, 5, 50 ng/ml) or the corresponding vehicle control. (A) RBP4 mRNA expression was measured by quantitative PCR. RBP4 expression was normalized to succinate dehydrogenase complex subunit A (SDHA) and related to medium control using 2^−ΔΔCT^ method. Data are presented as mean+SEM of three independent experiments. *p<0.05 (treatment vs. vehicle). (B) Accumulation of RBP4 in the medium supernatant was measured by ELISA. Measurements were related to untreated medium control. Data are presented as mean+SEM of three independent experiments. *p<0.05 (treatment vs. vehicle). (C, D) Human primary adipocytes obtained from three different donors were treated with 5 ng/ml of IL-1β or vehicle. (C) mRNA expression and (D) secretion of RBP4 was performed as described above.*p<0.05 (treatment vs. vehicle). (E) SGBS adipocytes were treated with 5 ng/ml of IL-1β for 24, 48, and 72 hours. mRNA expression of RBP4 was measured by quantitative PCR. RBP4 expression was normalized to SDHA and related to medium control at 0 hours using 2^−ΔΔCT^ method. *p<0.05 (treatment vs. vehicle).

We treated SGBS adipocytes with increasing doses of IL-1β (0.05–50 ng/ml) to study the dose-dependent effects of this cytokine on RBP4 production. With 0.05 ng/ml IL-1β we detected a downregulation to 69±7% of the vehicle after 48 hours and reached a maximal effect at 5 ng/ml (Figure 3A). Concentration of 0.05 ng/ml was not sufficient to significantly block RBP4 secretion, however with 5 ng/ml we detected a downregulation to 50±1% of the vehicle as detected by ELISA (Figure 3B). Comparable results were found with human primary *ex vivo* differentiated adipocytes obtained from three different donors (Figure 3C and D). The inhibition of RBP4 mRNA was time-dependent and most efficient after 72 hours (Figure 3E).

### Activation of NF-κB via IL-1R type 1 (IL-1R1) is Responsible for IL-1β Mediated Downregulation of RBP4

**Figure 4:**
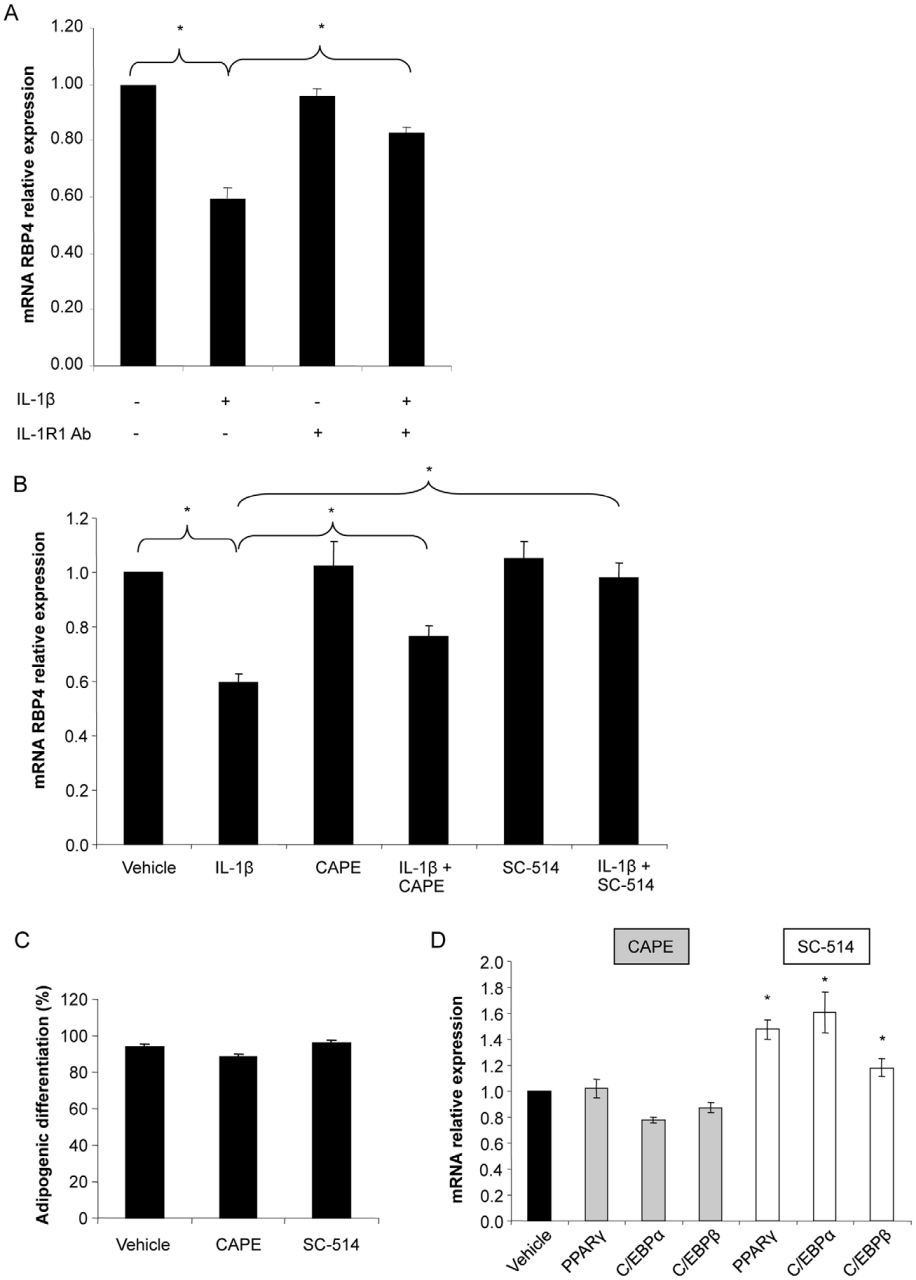
Inhibition of IL-1R1 and NF-κB pathway inhibitor blunted the inhibitory effect of IL-1β on RBP4 production. (A) SGBS adipocytes were treated with IL-1β (0.5 ng/ml) and/or IL-1 receptor type 1 specific neutralizing antibody (IL-1R1 Ab; 100 µg/ml) or the corresponding vehicle control. RBP4 mRNA expression was measured by quantitative PCR. RBP4 expression was normalized to succinate dehydrogenase complex subunit A (SDHA) and related to medium control using 2^−ΔΔCT^ method. Data are presented as mean+SEM of three independent experiments. *p<0.05 vehicle vs. IL-1β; ^#^p<0.05 IL-1β vs. IL-1β+IL-1R1 Ab. (B) SGBS adipocytes were treated with IL-1β (0.5 ng/ml) alone or in combination with NF-κB inhibitors CAPE (1 µg/ml) or SC-514 (100 µM) respectively. mRNA expression analysis of RBP4 was performed as described above. *p<0.05 vehicle vs. IL-1β; ^#^p<0.05 IL-1β vs. IL-1β+CAPE or IL-1β +SC-514. (C, D) SGBS cells were treated with CAPE (1 µg/ml) or SC-514 (100 µM) during the process of adipogenic differentiation. (C) The rate of adipogenic differentiation was determined on day 10. (D) mRNA expression of PPARγ, C/EBPα and C/EBPβ was determined by qPCR. *p<0.05 vehicle vs. SC-514.

We next aimed to identify the signaling mechanisms by which IL-1β mediates its effects on RBP4 production. First, we used an antagonistic antibody to block the function of the IL-1R1 (Figure 4A). The IL-1β stimulated decrease of RBP4 was reduced by neutralizing the function of IL-1R1 with a specific antibody demonstrating that this specific receptor mediates the effects of IL-1β on RBP4 expression. Activation of the IL-1 receptor results in activation of the NF-κB pathway [20]. To determine the role of NF-κB we used two inhibitors of this pathway, CAPE and SC-514. The IL-1β mediated downregulation of RBP4 was reduced by both CAPE and SC-514 (Figure 4B). In a control experiment we showed that neither CAPE nor SC-514 significantly affect rates of adipogenic differentiation (Fig. 4C). Furthermore, CAPE did not interfere with the expression of adipogenic marker genes PPARγ, C/EBPα, and C/EBPβ; SC-514, however, increased their expression (Figure 4D). Altogether, these data demonstrate that activation of NF-κB by IL-1β is necessary to mediate its effect on RBP4 expression.

### Negative Correlation between RBP4 and IL-1β Expression in Human Adipose Tissue

**Figure 5:**
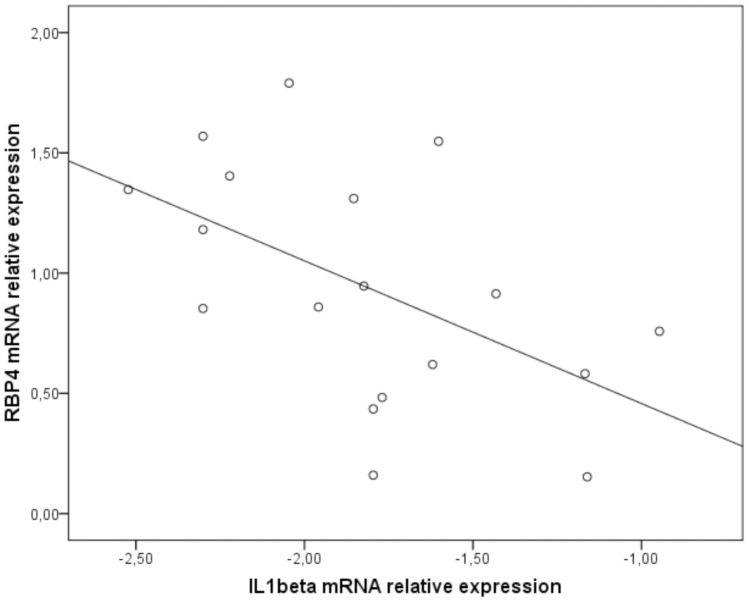
Negative correlation between the relative mRNA expression of RBP4 and IL-1β in human adipose tissue explants. RBP4 mRNA relative expression ratios in samples from subcutaneous adipose tissue of 18 healthy middle-aged females with a wide range of BMIs were determined by quantitative PCR and normalized to succinate dehydrogenase complex subunit A (SDHA) by 2^−ΔΔCT^ method. Due to a non-normal distribution, IL-1β mRNA relative expression ratios were subjected to logarithmic transformation. Correlation between RBP4 and IL-1β expression was determined by Pearson correlation coefficient (R = −0.535; p<0.05).

We next wanted to explore if IL-1β might be a putative regulator of RBP4 expression *in vivo*. We therefore studied the mRNA expression of RBP4 and markers of adipose tissue inflammation in subcutaneous adipose tissue from 18 healthy women. As expected from previous reports, we detected a positive association of RBP4 (R = 0.470; p<0.05) and also the macrophage marker CD68 with BMI (R = 0.472; p = 0.09). The mRNA expression of IL-1β was however not associated with BMI (R = −0.268) and expression of CD68 (R = −0.121). Most interestingly however and in line with our *in vitro* data, we detected a negative correlation between mRNA expression of RBP4 and IL-1β (R = −0.535; p<0.05) (Figure 5). This data underlines that local levels of IL-1β might cause a downregulation of RBP4 in human adipose tissue.

## Discussion

Excess adipose tissue accumulation is linked to the accumulation of inflammatory cells within adipose tissue, which is causally associated with the change in the secretion pattern of human adipocytes [4]. We show here that an inflammatory micro-environment significantly decreases RBP4 expression and secretion in human adipocytes.

In several mouse models as well as in humans, Graham et al. showed that a systemic increase in RBP4 precedes the onset of insulin resistance [21]. Adipose tissue inflammation is one of the first steps in the pathogenesis of insulin resistance [1]. Thus, we expected that an inflammatory milieu in adipose tissue would cause an increase in adipose tissue RBP4 and might therefore contribute to the increase in circulating RBP4 levels. On the opposite however, we found that a cocktail of factors secreted from macrophages, as well as the single factor IL-1β downregulated RBP4 mRNA expression and secretion from human adipocytes.

The knowledge on the hormonal regulation of RBP4 is still scarce. Rosiglitazone and adiponectin were identified as positive regulators, while TNF-α was described as a potent inhibitor of RBP4 production in human primary adipocytes [14]. In line with this, treatment of morphologically differentiated SGBS adipocytes with TNF-α resulted in a decrease in RBP4 expression [14]. MacCM robustly downregulated RBP4 and contained significant amounts of TNF-α suggesting this cytokine is a major regulator. In this study, we identified IL-1β as a new suppressor of RBP4 in human adipocytes (Figure 2). The effect was dose- and time-dependent and detectable on the mRNA and on the protein level. The clinical relevance of these observed effects is supported by the fact that they were achieved already with physiological concentrations of IL-1β [18], [22]. Interestingly, long term IL-1 treatment also decreases expression of adiponectin and leptin in human adipocytes [18], [23]. On the other hand IL-6 and IL-8 did not significantly change RBP4 expression in human adipocytes. As for IL-6, a cytokine known to be involved in the development of obesity complications, it is possible that the observed lack of effect is due to the fact that IL-6 soluble receptor was not added to IL-6 in our experiments. Namely it was previously determined that IL-6 decreases adiponectin expression only when used in combination with the soluble receptor [18]. Altogether, our results suggest IL-1β as an additional cytokine involved in the regulation of RBP4 expression in human adipocytes.

Additional, to date non-identified, macrophage-derived factors must however play a role in the described downregulation of RBP4. It is likely that a combination of factors has a more potent effect than single factors, since MacCM was more potent in downregulating RBP4 than single identified factors.

It was shown that MacCM in addition to promoting insulin resistance inhibits adipogenic differentiation in murine 3T3-L1 adipocytes and human adipocytes [19], [24]. This could be of significance in our case as RBP4 expression significantly increases during the process of adipogenic differentiation [7]. Namely, we also observe a previously described delipidating effect by inflammatory conditions in human adipocytes [18], [25]. Treatment with MacCM decreased adipocyte triglyceride content, while it increased glycerol concentration in the supernatant, indicating increased lipolysis. Altogether, the observed decrease in RBP4 expression in human adipocytes exposed to an inflammatory micro-environment could be directly associated with a decrease in adipogenic differentiation and delipidation of morphologically differentiated adipocytes.

Adipose tissue RBP4 expression positively correlates with BMI in certain studies, although it should be acknowledged that there is no or even negative correlation in others [12]. These differences are in part attributed to the variability in the study-population design (influence of ethnicity, gender, age, concomitant diseases) and shortcomings in the methodology (type of plasma anticoagulant in collection tube, immunoassay design). With regard to inflammation, RBP4 expression positively correlated with markers of macrophage infiltration in primary human adipose tissue explants [13]. The same trend was observed in our group of healthy middle-aged women with a wide range of BMIs. Interestingly, and in line with our *in vitro* data, a negative correlation between RBP4 and IL-1β mRNA expression was determined in subcutaneous adipose tissue from these women (Figure 5). Altogether, excess adipose tissue accumulation was associated with infiltration of pro-inflammatory macrophages, which resulted in a decreased RBP4 expression directly implicating inflammation in the decreased RBP4 expression.

The effects of IL-1β are mediated via the IL-1 receptor type I (IL-1R1) and the NF-κB pathway [26]. In our experiments, the effect of IL-1β was blunted by the blockade of the IL-1R1 and the NF-κB pathway (Figure 4A and B). These results provide a possible mechanism by which IL-1β mediates its effects on RBP4 expression, thereby linking inflammation with dysregulated adipokine secreting function of human adipocytes. It should however be stressed, that besides IL-1R1 other IL-1 receptors may have a role in mediating this effect, as the effect of IL-1β was not completely neutralized by IL-1R1 Ab (Figure 4A). The IL-1 family is strongly implicated in the pathogenesis of acute and chronic inflammation [19]. IL-1β is emerging as a therapeutic target in several inflammatory conditions, including type 2 diabetes [27]. Our results suggest that IL-1β blockade could have important effects on the endocrine function of adipocytes and consequently on the development of obesity-associated comorbidities.

In conclusion, the expression of an adipokine implicated in the development of whole body insulin resistance, RBP4, is downregulated by an inflammatory micro-environment in adipose tissue. In addition to the previously described effect of TNF-α, we determined IL-1β as an additional pro-inflammatory cytokine inhibiting RBP4 expression and secretion in human adipocytes. These results are corroborated by the finding of a negative correlation between RBP4 and IL-1β expression in the human adipose tissue explants.

Altogether our results implicate adipose tissue inflammation and specifically IL-1β in the regulation of RBP4 expression in human adipose tissue. The increase in circulating RBP4 that often precedes the development of systemic insulin resistance is most likely unrelated to these processes.
